# Supplementary material for: Dual Engram Architecture within a Single Striatal Cell Type Distinctly Controls Alcohol Relapse and Extinction
Source: bioRxiv. 2026 Jan 14:2026.01.13.699375. Preprint. [Version 1] doi: 10.64898/2026.01.13.699375 (PMC12879658; doi:10.64898/2026.01.13.699375)
Supplement: Supplement 1 [file NIHPP2026.01.13.699375v1-supplement-1.pdf]

bioRxiv preprint doi: https://doi.org/10.64898/2026.01.13.699375; this version posted January 14, 2026. The copyright holder for this preprint (which was not certified by peer review) is the author/funder, who has granted bioRxiv a license to display the preprint in perpetuity. It is made available under aCC-BY 4.0 International license.

Supplementary Figures and Figure Legends

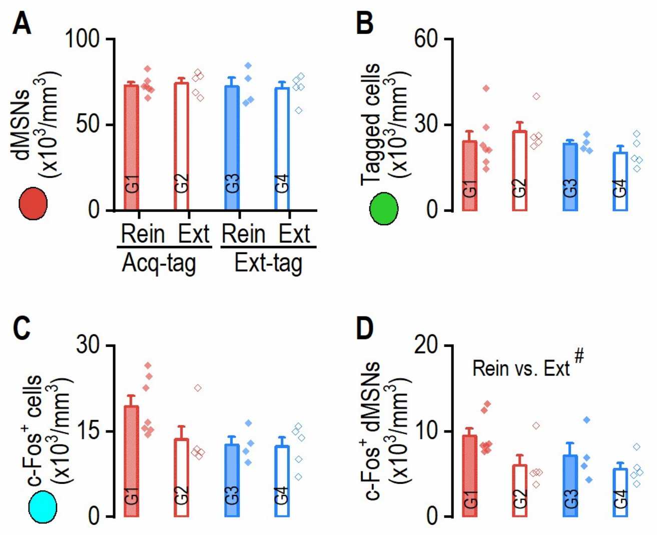

Supplementary Figure 1. Histological analysis of tagged dMSNs and c-Fos⁺ dMSNs in acquisition and extinction-tagged groups after extinction or reinstatement test (Related to Figure 1)

A, Density of dMSNs showed no difference among groups. Two-way ANOVA: Group × Test: $F_{(1, 17)} = 0.13$, $p = 0.73$.

B, Density of tagged cells had no difference among groups. Two-way ANOVA: Group × Test: $F_{(1, 17)} = 1.12$, $p = 0.31$.

C, Density of c-Fos⁺ cells had no difference among groups. Two-way ANOVA: Group × Test: $F_{(1, 17)} = 1.95$, $p = 0.18$.

D, Groups tested for cue-induced reinstatement showed a higher density of c-Fos⁺ dMSNs compared to groups tested for extinction. Two-way ANOVA: Test (Rein vs Ext): $F_{(1, 17)} = 5.52$, $^{\#}p = 0.031$.

n = 7 mice (Acq-tag, Rein test), 5 mice (Acq-tag, Ext test), 4 mice (Ext-tag, Rein test), and 5 mice (Ext-tag, Ext test).

bioRxiv preprint doi: https://doi.org/10.64898/2026.01.13.699375; this version posted January 14, 2026. The copyright holder for this preprint (which was not certified by peer review) is the author/funder, who has granted bioRxiv a license to display the preprint in perpetuity. It is made available under aCC-BY 4.0 International license.

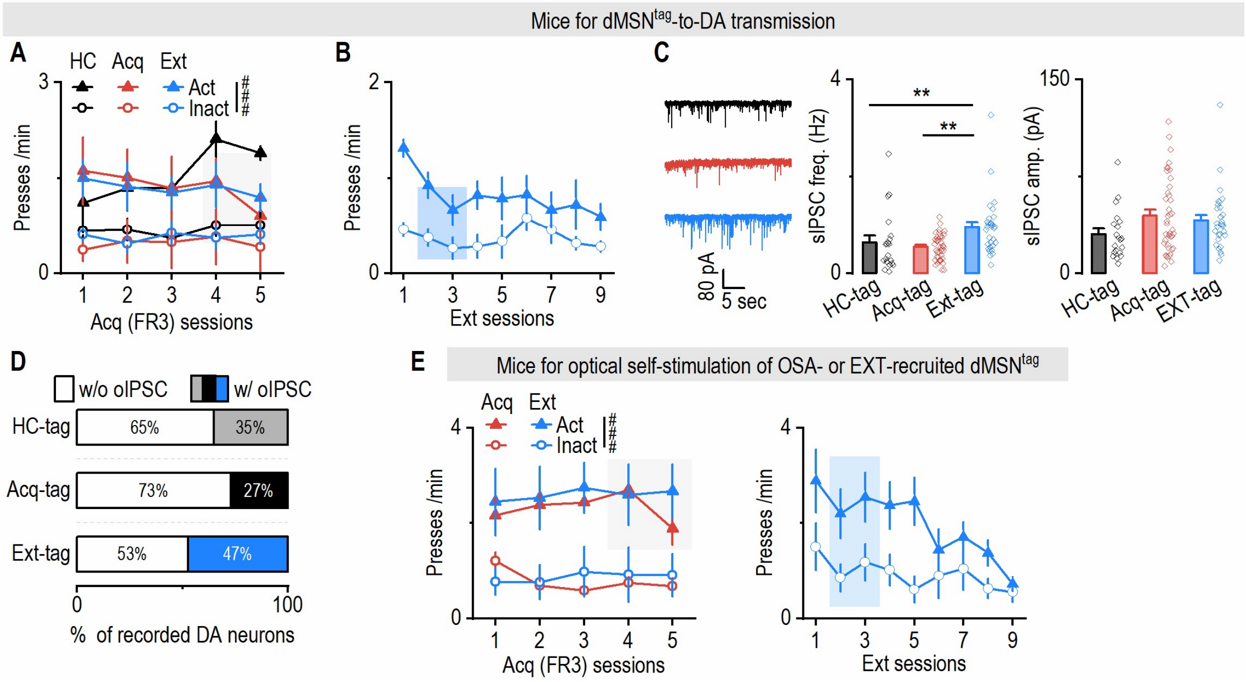

Supplementary Figure 2. Behavioral performance of mice used in Figure 2

A, Active presses were similar among the three groups (Two-way RM ANOVA: Group: $F_{(2,10)} = 0.12$, $p = 0.89$), and were higher than their inactive presses (Two-way RM ANOVA: Lever: $F_{(1,12)} = 46.78$, $^{###}p < 0.001$).

B, In the Ext group, active presses gradually decreased to the level of inactive presses over the course. Two-way RM ANOVA: Lever × Session: $F_{(8,32)} = 1.76$, $p = 0.12$. Sidak's multiple comparisons test: For sessions 1-9: t = 5.84, 3.78, 2.76, 3.69, 3.10, 1.69, 1.46, 2.78, 2.14, $p < 0.001$ for session 1, $p < 0.01$ for sessions 2 and 4, $p < 0.05$ for session 5, $p > 0.05$ for sessions 3 and 6-9.

C, SNc DA neurons exhibited similar sIPSC amplitudes across groups (Kruskal-Wallis test: $H_{(2)} = 4.73$, $p = 0.09$).

D, A greater percentage of recorded SNc DA neurons received oIPSCs from dMSN^{tag} in the Ext-tag group to the HC-tag and Acq-tag groups. Chi-square test: $X^2_{(2)} = 3.64$, $p = 0.16$.

bioRxiv preprint doi: https://doi.org/10.64898/2026.01.13.699375; this version posted January 14, 2026. The copyright holder for this preprint (which was not certified by peer review) is the author/funder, who has granted bioRxiv a license to display the preprint in perpetuity. It is made available under aCC-BY 4.0 International license.

E, For mice tested for oICSS, active presses during the acquisition phase (left) were similar between groups (Three-way ANOVA: Group: $F_{(1,8)} = 0.17$, $p = 0.69$), and exceeded the level of inactive presses (Three-way ANOVA: Lever: $F_{(1,8)} = 29.03$, $^{###}p < 0.001$). During extinction (middle), active presses progressively declined to the level of inactive presses over the course (Two-way RM ANOVA: Lever × Session: $F_{(8,32)} = 4.05$, $p = 0.002$. Sidak's multiple comparisons test: For sessions 1-9: $t = 5.19, 5.04, 5.12, 5.08, 6.94, 2.03, 2.51, 2.81, 0.63$, $p < 0.001$ for sessions 1-5, $p > 0.05$ for sessions 6-9). The inactive presses (right) during the last acquisition session (Acq-tag) and the last extinction session (Ext-tag) were comparable (Unpaired t test: $t_{(7)} = 0.102$, $p = 0.92$).

n = 3 mice (A, B; HC-tag), 5 mice per group (A, B; Acq-tag and Ext-tag), 20 neurons from 3 mice (20/3; HC-tag; C), 40/5 (Acq-tag; C), 30/5 (Ext-tag; C), 20/3 (HC-tag; D), 48/5 (Acq-tag; D), 36/5 (Ext-tag; D), 4 mice (Acq-tag; E) and 5 mice (Ext-tag; E).

bioRxiv preprint doi: https://doi.org/10.64898/2026.01.13.699375; this version posted January 14, 2026. The copyright holder for this preprint (which was not certified by peer review) is the author/funder, who has granted bioRxiv a license to display the preprint in perpetuity. It is made available under aCC-BY 4.0 International license.

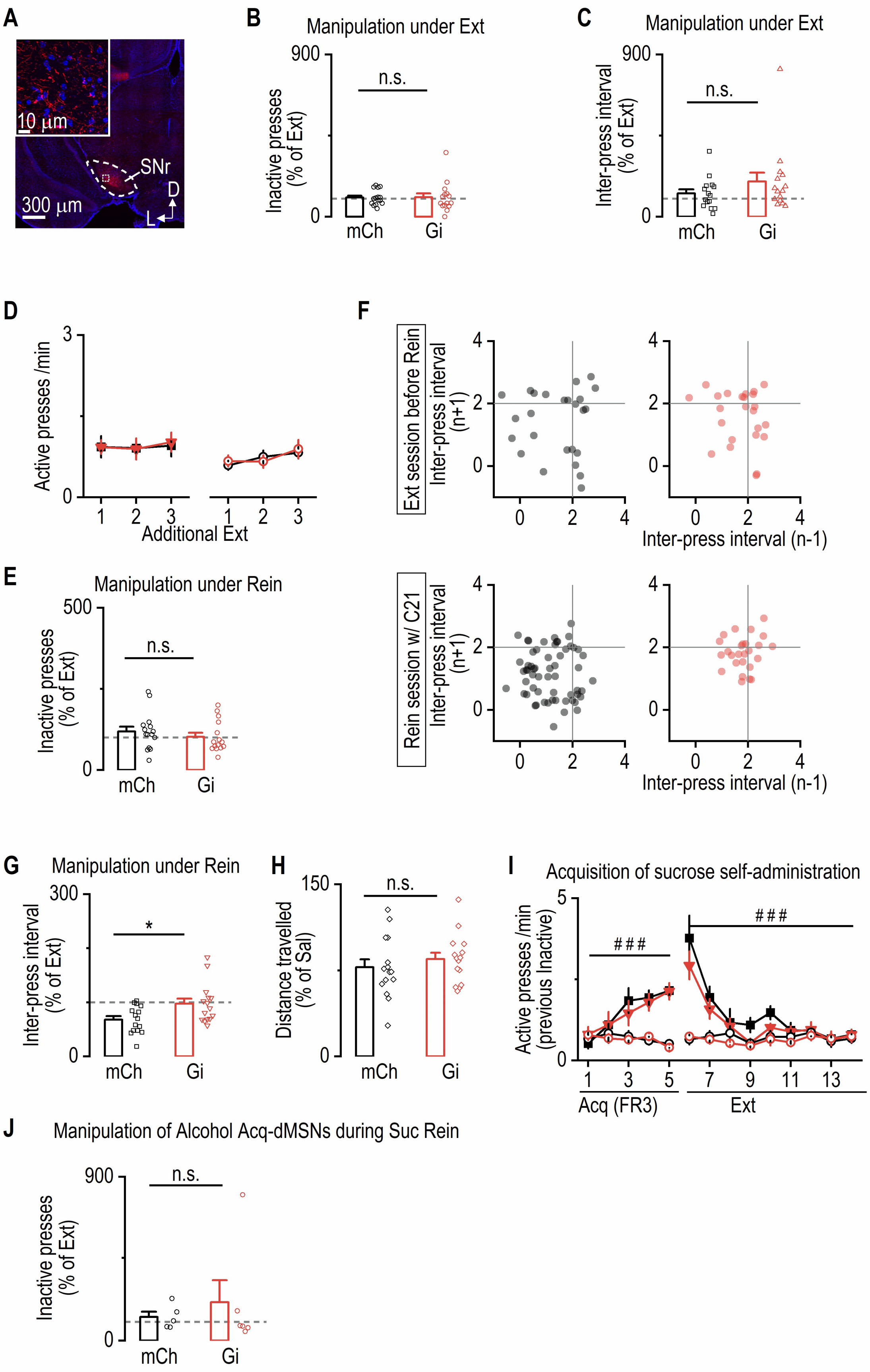

bioRxiv preprint doi: https://doi.org/10.64898/2026.01.13.699375; this version posted January 14, 2026. The copyright holder for this preprint (which was not certified by peer review) is the author/funder, who has granted bioRxiv a license to display the preprint in perpetuity. It is made available under aCC-BY 4.0 International license.

Supplementary Figure 3. Other behavioral parameters during chemogenetic inhibition of alcohol acquisition-recruited dMSNs (Related to Figure 3)

A, Sample confocal images showing hM4Di-mCherry$^+$ fibers, but not cell bodies, in the SNr. D, dorsal; L, lateral.

B, Chemogenetic inhibition of Acq-recruited dMSNs during extinction did not alter inactive-pressing. Mann-Whitney test: U = 101, p = 0.653.

C, Chemogenetic inhibition of alcohol acquisition-recruited dMSNs during the extinction test did not alter the inter-press interval for active lever between groups. Mann-Whitney test: U = 84, p = 0.25.

D, Both groups exhibited similar levels of active and inactive presses during additional extinction sessions. Three-way ANOVA: Group: $F_{(1,28)} = 0.01$, $p = 0.91$.

E, Chemogenetic inhibition of Acq-recruited dMSNs during cue-induced reinstatement did not alter inactive pressing compared to the mCh group. Mann-Whitney test: U = 94, p = 0.455.

F, G, Sample inter-press intervals analyzed from the last additional extinction session (F, top) and cue-induced reinstatement (F, bottom) from one mouse in each group. Chemogenetic inhibition of acquisition-recruited dMSNs increased inter-press intervals compared to the mCh group (G: unpaired t test: $t_{(28)} = 2.56$, $^*p = 0.016$).

H, Chemogenetic manipulation of alcohol-recruited dMSNs did not affect the distance traveled in an open field test. Unpaired t test: $t_{(28)} = 0.79$, $p = 0.43$.

I, Both groups showed similar increases in active presses during 5 d of sucrose training (Three-way ANOVA: Lever × Session: $F_{(4,36)} = 16.06$, $^{\#\#\#}p < 0.001$), followed by significant

bioRxiv preprint doi: https://doi.org/10.64898/2026.01.13.699375; this version posted January 14, 2026. The copyright holder for this preprint (which was not certified by peer review) is the author/funder, who has granted bioRxiv a license to display the preprint in perpetuity. It is made available under aCC-BY 4.0 International license.

reductions during extinction (Three-way ANOVA: Lever × Session: $F_{(8,72)} = 31.91$, $^{\#\#\#}p < 0.001$).

J, Chemogenetic inhibition of alcohol acquisition-recruited dMSNs did not affect inactive presses during cue-induced reinstatement of sucrose seeking. Mann-Whiteney test: $U = 13$, $p = 0.79$.

n = 15 mice per group (B-E, G, H), 5 mice (**I**, J; mCh), 6 mice (**I**, J; Gi).

bioRxiv preprint doi: https://doi.org/10.64898/2026.01.13.699375; this version posted January 14, 2026. The copyright holder for this preprint (which was not certified by peer review) is the author/funder, who has granted bioRxiv a license to display the preprint in perpetuity. It is made available under aCC-BY 4.0 International license.

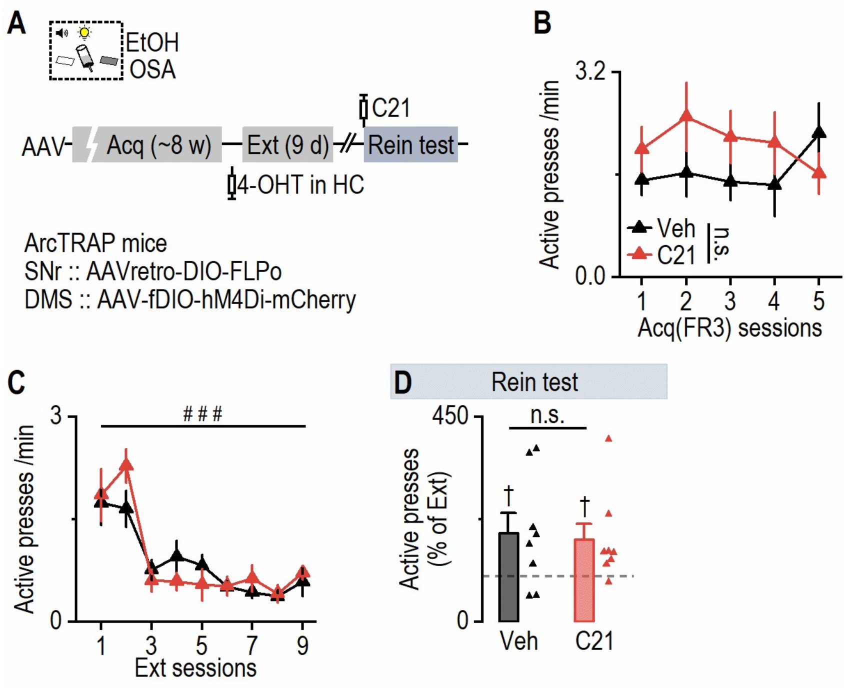

Supplementary Figure 4. Chemogenetic inhibition of home-cage-recruited dMSNs did not mediate a cue-alcohol memory retrieval (Related to Figure 3)

A, Schematic of the experiment timeline. ArcTRAP mice were infused with AAVretro-DIO-FLPo into the SNr and AAV-fDIO-hM4Di-mCherry into the DMS. Mice were trained for EtOH OSA for ~8 weeks. At least 24 h after the last OSA session, mice were administered 4-OHT in the home-cage for two consecutive days. One week later, mice underwent 9 d of extinction training, followed by a cue-induced reinstatement test with C21 pre-injection.

B, Active presses were similar between groups. Two-way RM ANOVA: Group: $F_{(1,14)}$ = 0.74, p = 0.40.

C, Extinction training significantly reduced active presses in both groups. Two-way RM ANOVA: Session: $F_{(8,112)}$ = 20.1, $^{###}p < 0.001$.

D, Chemogenetic inhibition of home-cage-recruited dMSNs did not alter cue-induced reinstatement of alcohol seeking. Compared to 100% theoretical baseline: one-sample t

bioRxiv preprint doi: https://doi.org/10.64898/2026.01.13.699375; this version posted January 14, 2026. The copyright holder for this preprint (which was not certified by peer review) is the author/funder, who has granted bioRxiv a license to display the preprint in perpetuity. It is made available under aCC-BY 4.0 International license.

test: Veh: $t_{(7)} = 3.06$, $^{\dagger}p = 0.01$; C21: $t_{(7)} = 2.32$, $^{\dagger}p = 0.053$. Group comparison: Mann-Whitney test: $U = 23$, $p = 0.38$.

n = 8 mice per group.

bioRxiv preprint doi: https://doi.org/10.64898/2026.01.13.699375; this version posted January 14, 2026. The copyright holder for this preprint (which was not certified by peer review) is the author/funder, who has granted bioRxiv a license to display the preprint in perpetuity. It is made available under aCC-BY 4.0 International license.

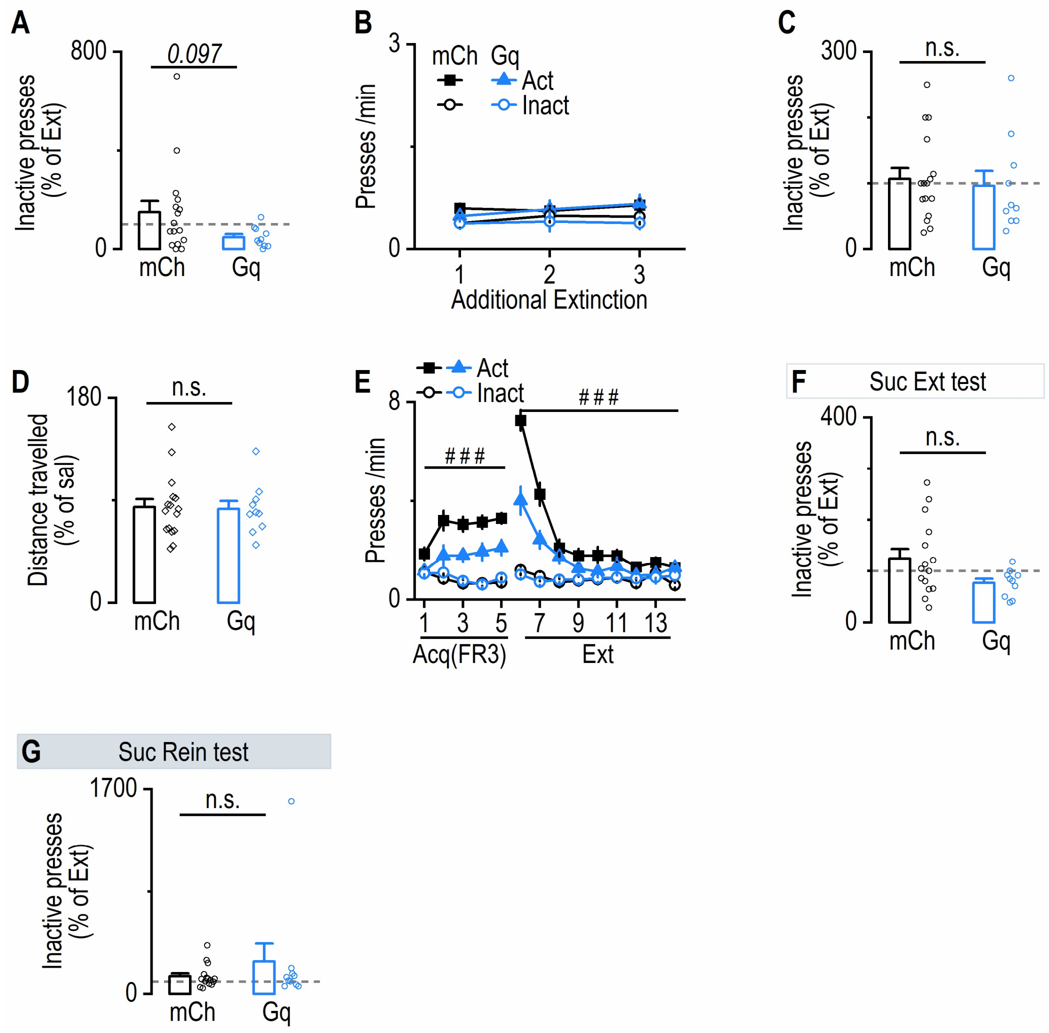

Supplementary Figure 5. Additional behavior and locomotor activity of mice used in Figure 4

A, During the extinction test, activation of extinction-recruited dMSNs marginally reduced normalized inactive presses compared to mCh. Mann-Whitney test: $U = 48$, $p = 0.097$.

B, Both groups showed similar active and inactive presses during 3 d of additional extinction training. Three-way ANOVA: Group: $F_{(1,24)} = 0.18$, $p = 0.68$.

C, Activation of extinction-recruited dMSNs did not affect inactive presses during the reinstatement test. Mann-Whitney test: $U = 67.5$, $p = 0.52$.

bioRxiv preprint doi: https://doi.org/10.64898/2026.01.13.699375; this version posted January 14, 2026. The copyright holder for this preprint (which was not certified by peer review) is the author/funder, who has granted bioRxiv a license to display the preprint in perpetuity. It is made available under aCC-BY 4.0 International license.

D, Chemogenetic activation of extinction-recruited dMSNs did not alter locomotor activity compared to the mCh group. Mann-Whitney test: $U = 79$, $p = 0.979$.

E, Both groups successfully acquired sucrose self-administration, as indicated by significantly higher active versus inactive lever presses across the 5-day training period (Mixed-effect analysis: Session × Lever: $F_{(4,90)} = 15.77$, $^{\#\#\#}p < 0.001$. Session × Group: $F_{(4,92)} = 0.28$, $p = 0.89$). During extinction training, active lever presses declined in both groups (three-way ANOVA: Session × Lever: $F_{(8,184)} = 59.06$, $^{\#\#\#}p < 0.001$).

F, Chemogenetic activation of EtOH extinction-recruited dMSNs did not alter inactive presses during sucrose extinction test. Unpaired t test: $t_{(23)} = 1.97$, $p = 0.06$.

G, Chemogenetic activation of EtOH extinction-recruited dMSNs did not alter inactive presses during sucrose-cue-induced reinstatement test. Mann-Whitny test: $U = 72$, $p = 0.89$.

n = 16 mice (mCh; A-D), 10 mice (Gq; A-D), 15 mice (mCh; E-F) and 10 mice (Gq; E-F).

bioRxiv preprint doi: https://doi.org/10.64898/2026.01.13.699375; this version posted January 14, 2026. The copyright holder for this preprint (which was not certified by peer review) is the author/funder, who has granted bioRxiv a license to display the preprint in perpetuity. It is made available under aCC-BY 4.0 International license.

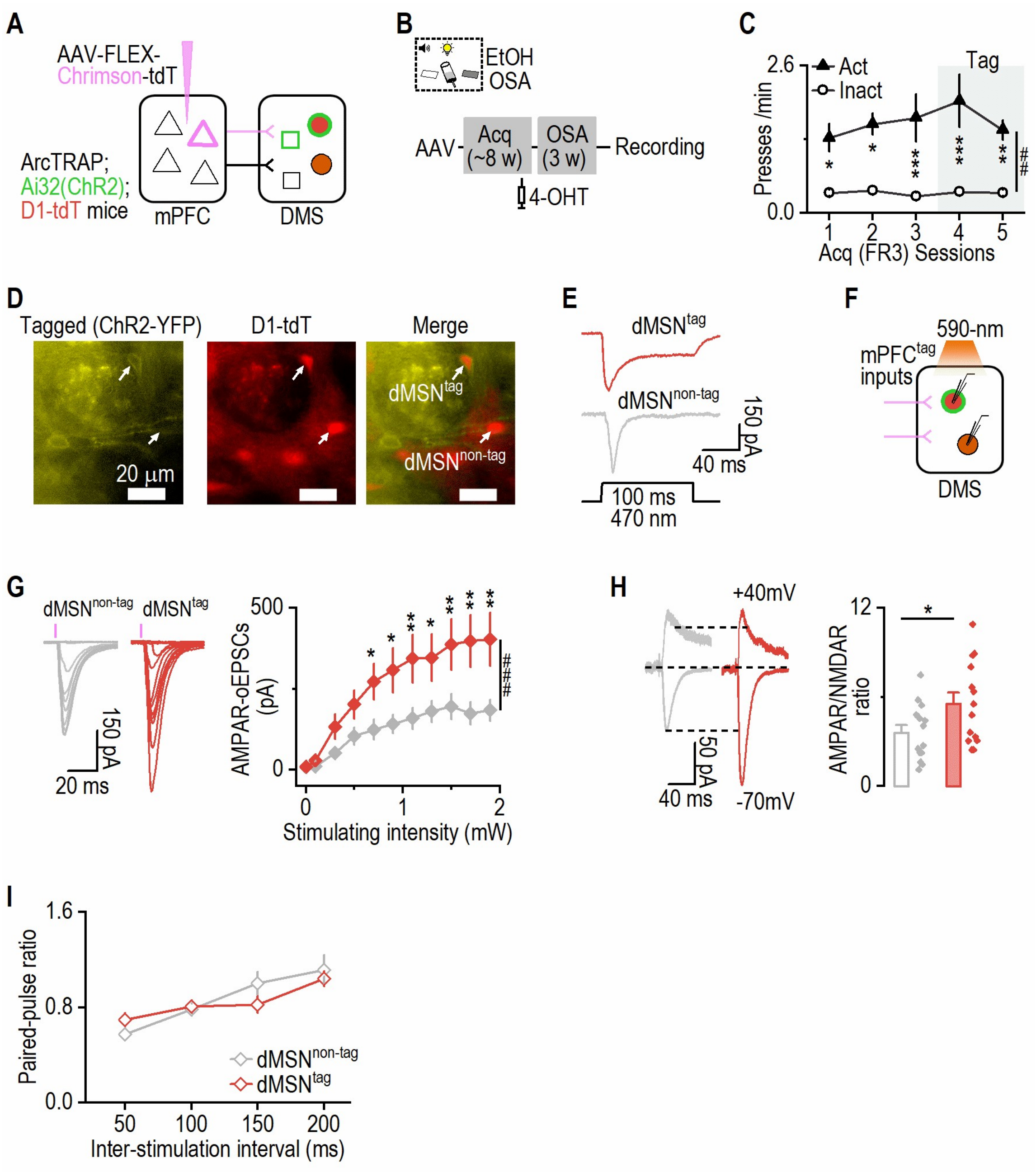

Supplementary Figure 6. mPFC^{tag}-to-dMSN^{tag} transmission, and paired-pulse ratio in ArcTRAP;Ai32;D1-tdT mice (Related to Figure 5)

A, Schematic showing ChR2-EYFP and Chrimson-tdT expression in tagged corticostriatal synapses of ArcTRAP;Ai32 (ChR2-EYFP);D1-tdT mice. AAV-FLEX-Chrimson-tdT was infused into the mPFC to measure optically-induced excitatory

bioRxiv preprint doi: https://doi.org/10.64898/2026.01.13.699375; this version posted January 14, 2026. The copyright holder for this preprint (which was not certified by peer review) is the author/funder, who has granted bioRxiv a license to display the preprint in perpetuity. It is made available under aCC-BY 4.0 International license.

postsynaptic currents (oEPSCs) from tagged mPFC neurons ($mPFC^{tag}$) to tagged dMSNs ($dMSN^{tag}$) versus non-tagged dMSNs ($dMSN^{non\text{-}tag}$). In the DMS, circle represents dMSN and square represents non-dMSN.

B, EtOH acquisition-activated neurons were tagged during two FR3 sessions, training continued for 3 weeks, and mice were sacrificed 1 d after the last training for electrophysiology.

C, Comparison of active and inactive presses during training and tagging sessions. Mixed-effect analysis: Act vs. Inact: $F_{(1, 12)} = 16.73$, $^{\#\#}p = 0.0015$. Sessions 1-5: $t = 2.86$, 3.05, 4.07, 4.72, 3.26, $^{*}p < 0.05$ (1, 2), $^{***}p < 0.001$ (3, 4), and $^{**}p < 0.01$ (5).

D, Images acquired during electrophysiology recording showing a visually identified $dMSN^{tag}$ (co-expressing ChR2-EYFP and tdT) and a nearby $dMSN^{non\text{-}tag}$ (tdT only).

E, Sample traces demonstrate direct depolarization in the ChR2-expressing $dMSN^{tag}$, while the $dMSN^{non\text{-}tag}$ shows only oEPSCs in response to 100 ms blue light stimulation.

F, Schematic of synaptic transmission recording from $mPFC^{tag}$ to DMS $dMSN^{tag}$ or $dMSN^{non\text{-}tag}$ under 590 nm light stimulation.

G, AMPA receptor-mediated oEPSCs (AMPAR-oEPSCs) from acquisition-activated mPFC inputs were stronger in $dMSN^{tag}$ than $dMSN^{non\text{-}tag}$. Mixed-effect analysis: Group × Intensity: $F_{(10, 340)} = 5.23$, $^{\#\#\#}p < 0.001$; $^{*}p < 0.05$, $^{**}p < 0.01$ as compared to $dMSN^{non\text{-}tag}$ group under the same stimulation intensity.

H, AMPAR/NMDAR ratios (measured with oEPSCs from acquisition-activated mPFC inputs) were greater in $dMSN^{tag}$ compared to $dMSN^{non\text{-}tag}$. Unpaired t test: $t_{(26)} = 2.19$, $^{*}p = 0.038$.

bioRxiv preprint doi: https://doi.org/10.64898/2026.01.13.699375; this version posted January 14, 2026. The copyright holder for this preprint (which was not certified by peer review) is the author/funder, who has granted bioRxiv a license to display the preprint in perpetuity. It is made available under aCC-BY 4.0 International license.

I, The paired-pulse ratio (measured by stimulating EtOH acquisition-activated mPFC inputs) did not differ between $dMSN^{non-tag}$ and $dMSN^{tag}$. Two-way RM ANOVA: Group: $F_{(1, 34)} = 0.16$, $p = 0.69$.

n = 7 mice (C), 19 neurons from 7 mice (19/7; $dMSN^{non-tag}$; G), 17/7 ($dMSN^{tag}$; G), 14/7 per group (H), 18/7 per group (I).

bioRxiv preprint doi: https://doi.org/10.64898/2026.01.13.699375; this version posted January 14, 2026. The copyright holder for this preprint (which was not certified by peer review) is the author/funder, who has granted bioRxiv a license to display the preprint in perpetuity. It is made available under aCC-BY 4.0 International license.

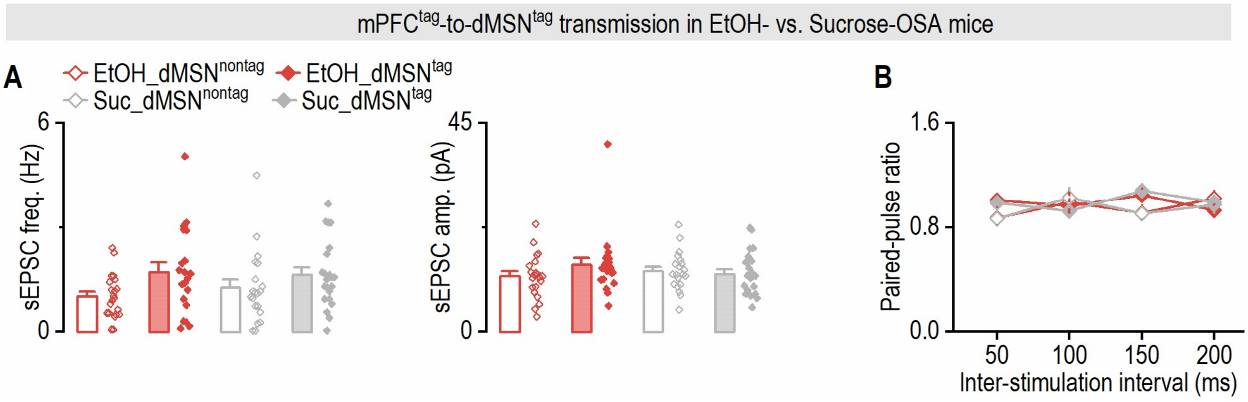

Supplementary Figure 7. Spontaneous excitatory synaptic transmission and paired-pulse ratio in alcohol- versus sucrose-trained mice (Related to Figure 5)

A, The frequency (left; two-way ANOVA: $F_{(1,79)}$ = 0.54, p = 0.466) and amplitude (right; two-way ANOVA: $F_{(1,79)}$ = 1.79, p = 0.18) of sEPSCs did not differ between dMSN$^{non\text{-}tag}$ and dMSN^{tag} in either the EtOH or sucrose groups.

B, The paired-pulse ratio (measured by stimulating EtOH or sucrose acquisition-activated mPFC inputs) did not differ between dMSN$^{non\text{-}tag}$ and dMSN^{tag}. Mixed-effect analysis: Substance × Tag: $F_{(1,\ 107)}$ = 0.058, p = 0.81.

n = 21 neurons from 5 mice (21/5; EtOH_dMSN$^{non\text{-}tag}$; A), 20/5 (EtOH_dMSN^{tag}; A), 20/5 (Suc_dMSN$^{non\text{-}tag}$; A), 22/5 (Suc_dMSN^{tag}; A), 20/5 (EtOH_dMSN$^{non\text{-}tag}$, EtOH_dMSN^{tag}, Suc_dMSN$^{non\text{-}tag}$; B), and 22/5 (Suc_dMSN^{tag}; B).

bioRxiv preprint doi: https://doi.org/10.64898/2026.01.13.699375; this version posted January 14, 2026. The copyright holder for this preprint (which was not certified by peer review) is the author/funder, who has granted bioRxiv a license to display the preprint in perpetuity. It is made available under aCC-BY 4.0 International license.

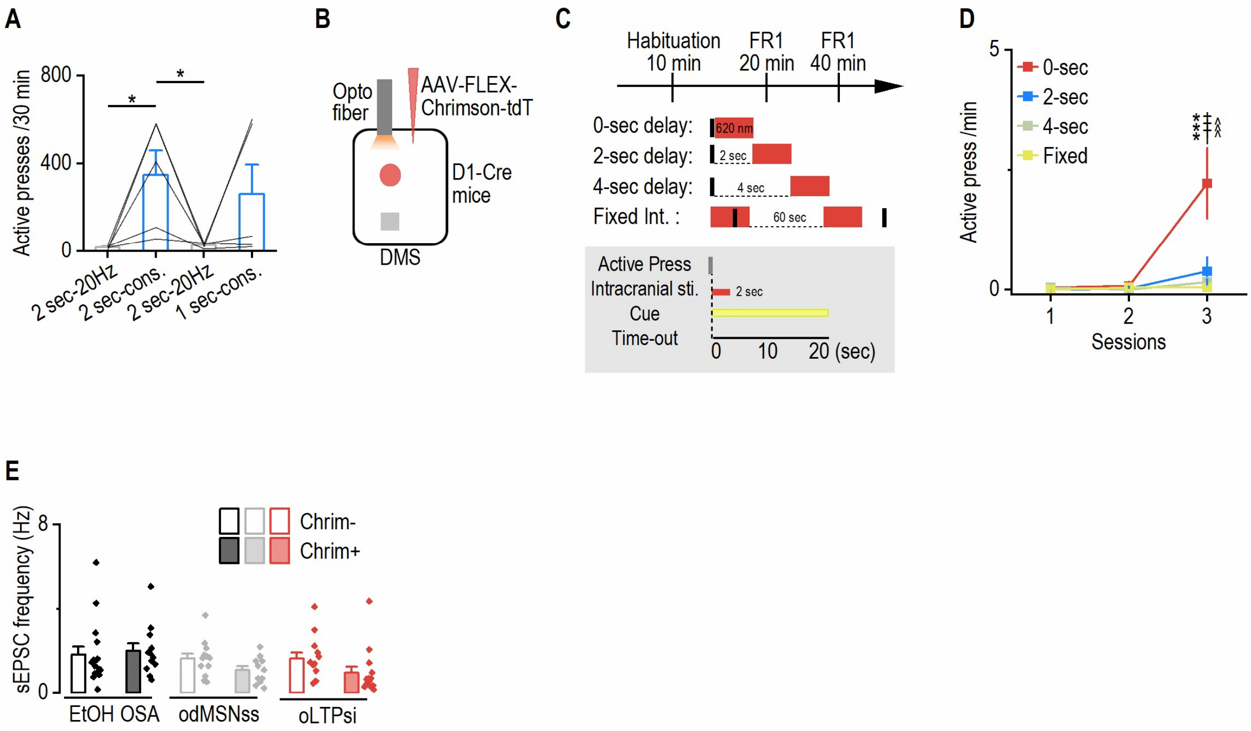

Supplementary Figure 8. The effect of different odMSNss protocols on operant lever-pressing (Related to Figure 6)

A, Control experiments were performed to assess potential optical bleed-through between opsins. Group 1: Wild-type mice received AAV-Chronos-GFP infusions into the mPFC with optic fibers implanted above the DMS. During training, these mice received the postsynaptic stimulation protocol using 590-nm constant light, the wavelength used in Figure 6 to depolarize dMSNs, which should not activate Chronos. Group 2: D1-Cre mice received AAV-FLEX-Chrimson-tdT infusions into the DMS with optic fibers implanted above the DMS. During training, these mice received the presynaptic stimulation protocol using 473-nm, 50-Hz light, the wavelength used in Figure 6 to activate Chronos-expressing mPFC terminals, which may activate Chrimson but to a much less extent. Both groups underwent one day of mock induction (habituation; no light) followed

bioRxiv preprint doi: https://doi.org/10.64898/2026.01.13.699375; this version posted January 14, 2026. The copyright holder for this preprint (which was not certified by peer review) is the author/funder, who has granted bioRxiv a license to display the preprint in perpetuity. It is made available under aCC-BY 4.0 International license.

by 3 d of FR1 self-stimulation training, in which each active press triggered the same light-cue stimulation protocol used in Figure 6.

B, Neither inappropriate wavelength produced self-stimulation behavior. Specifically, 590-nm light failed to drive self-stimulation in Chronos-expressing mice, and 473-nm light failed to drive self-stimulation in Chrimson-expressing mice, demonstrating minimal functional cross-activation under our experimental conditions. Three-way ANOVA: Lever × Session: $F_{(3, 24)} = 0.55$, $p = 0.65$. n = 5 mice per group.

C, Three different stimulation protocols for odMSNss were tested: 2-sec 20-Hz pulsed light, 2-sec constant light, and 1-sec constant light. Only 2-sec constant light stimulation caused an increase in active presses. One-way RM ANOVA: $F_{(3, 12)} = 4.99$, $p = 0.018$. Tukey's multiple comparisons test: 2-sec 20 Hz vs. 2-sec constant: $q = 4.42$, $^{*}p = 0.038$; 2-sec constant vs. 2-sec 20 Hz again: $q = 4.31$, $^{*}p = 0.044$. n = 4 rats.

D, Schematic illustrating AAV-FLEX-Chrimson-tdT infusion into the DMS of D1-Cre mice with bilateral fiber implantation above the infusion sites.

E, After one day of habituation, different stimulation protocols for odMSNss were tested across 2 d of FR1: 0-sec delay (light stimulation immediately following an active press), 2-sec delay (2-sec delay between the active press and light stimulation), 4-sec delay, and fixed interval stimulation (light delivered every 60 sec, regardless of active presses).

F, Only the 0-sec delay group exhibited self-stimulation after 2 d of training. Two-way RM ANOVA: Protocol × Session: $F_{(6,12)} = 4.21$, $p = 0.016$. Tukey's multiple comparisons test: In session 3: 0-sec vs. 2-sec: $q = 6.93$, $^{***}p < 0.001$; 0-sec vs. 4-sec: $q = 6.94$, $^{\dagger\dagger\dagger}p < 0.001$; 0-sec vs. fixed interval: $q = 7.41$, $^{\wedge\wedge\wedge}p < 0.001$. n = 3 mice per group (0-sec and 2-sec), 2 mice per group (4-sec and fixed interval).

bioRxiv preprint doi: https://doi.org/10.64898/2026.01.13.699375; this version posted January 14, 2026. The copyright holder for this preprint (which was not certified by peer review) is the author/funder, who has granted bioRxiv a license to display the preprint in perpetuity. It is made available under aCC-BY 4.0 International license.

G, sEPSC frequencies did not differ between $Chrim^-$ and $Chrim^+$ neurons across all three groups. Two-way ANOVA: Group × Neuron ($Chrim^-$ vs. $Chrim^+$): $F_{(2,73)} = 2.47$, $p = 0.32$. n = 16 neurons/4 mice ($Chrim^-$ in EtOH), 12/4 ($Chrim^+$ in EtOH), 13/3 ($Chrim^-$ in odMSNss), 11/3 ($Chrim^+$ in odMSNss), 13/3 ($Chrim^-$ in oLTPsi), 14/3 ($Chrim^+$ in oLTPsi).

bioRxiv preprint doi: https://doi.org/10.64898/2026.01.13.699375; this version posted January 14, 2026. The copyright holder for this preprint (which was not certified by peer review) is the author/funder, who has granted bioRxiv a license to display the preprint in perpetuity. It is made available under aCC-BY 4.0 International license.

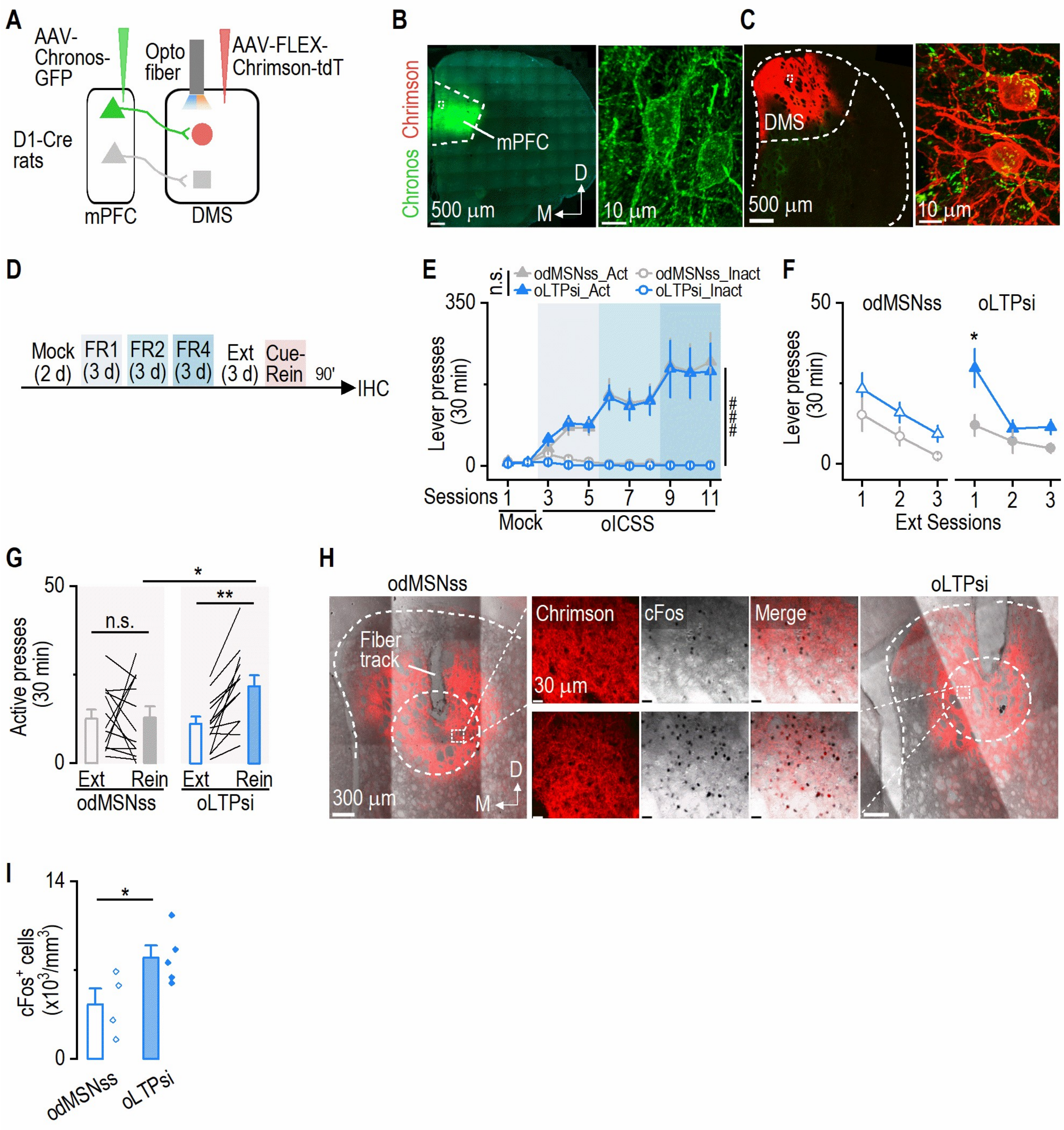

Supplementary Figure 9. oLTPsi and odMSNss in D1-Cre rats (Related to Figure 6)

A, The schematic of virus infusion and fiber implantation in D1-Cre rats.

B, C, Sample images showing Chronos-GFP expression in the mPFC (B) and Chrimson-tdT expression in the DMS (C). Enlarged images (right) showed individual neurons from the outlined regions (left). D, dorsal; M, medial.

bioRxiv preprint doi: https://doi.org/10.64898/2026.01.13.699375; this version posted January 14, 2026. The copyright holder for this preprint (which was not certified by peer review) is the author/funder, who has granted bioRxiv a license to display the preprint in perpetuity. It is made available under aCC-BY 4.0 International license.

D, Timeline for optogenetic intracranial self-stimulation (oICSS) using either oLTPsi or odMSNss.

E, Active and inactive presses during the training period were similar between odMSNss and oLTPsi groups (Three-way ANOVA: Group: $F_{(1,48)} = 0.03$, $p = 0.86$). Both groups performed more active presses than inactive presses (Three-way ANOVA: Lever: $F_{(1,48)} = 34.34$, $^{###}p < 0.001$).

F, The oLTPsi group, but not the odMSNss group, continued to display more active than inactive presses during the first extinction session. Three-way ANOVA: Group × Lever × Session: $F_{(2,96)} = 1.65$, $p = 0.19$. Tukey's multiple comparisons test: Act vs. Inact during first extinction in oLTPsi: $q = 4.98$, $^{*}p = 0.027$; Act vs. Inact during first extinction in odMSNss: $q = 2.23$, $p = 0.91$.

G, The oLTPsi group, but not the odMSNss group, showed cue-induced reinstatement of active pressing. Two-way RM ANOVA: Group × Session: $F_{(1,24)} = 6.07$, $p = 0.021$. Sidak's multiple comparisons test: Rein vs. Ext: $t = 0.10$, $p = 0.99$ (odMSNss); $t = 3.59$, $^{**}p = 0.003$ (oLTPsi). odMSNss vs. oLTPsi in Rein: $t = 2.37$, $^{*}p = 0.043$.

H, Sample confocal images of striatal sections from an odMSNss and an oLTPsi rat containing c-Fos^{+} neurons in the Chrimson-expressing region below the fiber track. Dashed circles indicate regions of analysis and dashed rectangles indicate where the enlarged images were taken from.

I, The oLTPsi group had more c-Fos^{+} neurons than the odMSNss group in the DMS regions with high Chrimson-tdT expression. $t_{(7)} = 2.40$, $^{*}p = 0.047$.

n = 13 rats/group (E-G), 4 rats (I; odMSNss), and 5 rats (I; oLTPsi).

bioRxiv preprint doi: https://doi.org/10.64898/2026.01.13.699375; this version posted January 14, 2026. The copyright holder for this preprint (which was not certified by peer review) is the author/funder, who has granted bioRxiv a license to display the preprint in perpetuity. It is made available under aCC-BY 4.0 International license.

Supplementary Information

List of acronyms used in the main text (in alphabetical order):

2BC – Two-Bottle Choice

4-OHT – 4-Hydroxytamoxifen

AAV – Adeno-Associated Virus

AMPA – α-amino-3-hydroxy-5-methyl-4-isoxazolepropionic acid

C21 – Compound 21 (a DREADD agonist)

DMS – Dorsomedial Striatum

dMSNs – Direct-Pathway Medium Spiny Neurons

DREADDs – Designer Receptors Exclusively Activated by Designer Drugs

EPSCs – Excitatory Postsynaptic Currents

FR – Fixed Ratio

Gi – Inhibitory hM_4D (Gi)

Gq – Excitatory hM_3D (Gq)

IHC – Immunohistochemistry

IPSCs – Inhibitory Postsynaptic Currents

LTP – Long-Term Potentiation

mCh – mCherry (fluorescent protein control)

MOR – Mu-Opioid Receptor

mPFC – Medial Prefrontal Cortex

NMDA – N-methyl-D-aspartate

oICSS – Optogenetic Intracranial Self-Stimulation

oLTPsi – Self-Induce Optical mPFC-to-dMSN Long-Term Potentiation

bioRxiv preprint doi: https://doi.org/10.64898/2026.01.13.699375; this version posted January 14, 2026. The copyright holder for this preprint (which was not certified by peer review) is the author/funder, who has granted bioRxiv a license to display the preprint in perpetuity. It is made available under aCC-BY 4.0 International license.

odMSNss – Optical dMSN Self-Stimulation

SNc – Substantia Nigra Pars Compacta

SNr – Substantia Nigra Pars Reticulata

tdT – tdTomato (fluorescent protein)

TRAP – Targeted Recombination in Active Populations

WD – Withdrawal
